# Availability of diagnostic tools and stewardship protocols for managing infectious diseases in Italian paediatric emergency departments: a nationwide survey

**DOI:** 10.1186/s13052-026-02197-7

**Published:** 2026-03-21

**Authors:** Marco Denina, Giulia Mazzetti, Emanuele Castagno, Stefania Zampogna, Claudia Bondone, SIMEUP Research Group

**Affiliations:** 1 Paediatric Infectious Diseases Unit, Regina Margherita Children’s Hospital, A.O.U. Città della Salute e della Scienza di Torino, Turin, Italy; 2 Department of Pediatrics and Public Health Sciences, Regina Margherita Children’s Hospital, Città della Salute e della Scienza, University of Turin, Turin, Italy; 3 Department of Paediatric Emergency, Regina Margherita Children’s Hospital, A.O.U. Città della Salute e della Scienza di Torino, Turin, Italy; 4 Paediatric Unit, Crotone Hospital, SIMEUP (Italian Society of Pediatric Emergency Medicine), Crotone, Italy

**Keywords:** Biomarkers of infections, Point of care technology, Antimicrobial stewardship, Paediatric emergency department, Fever, Children, Infectious diseases

## Abstract

**Background:**

Infectious diseases are a leading cause of Paediatric Emergency Department (PED) visits worldwide, characterised by significant clinical variability in both presentation and severity. Literature highlights that infection biomarkers and rapid etiological tests, when integrated into standardised clinical protocols, are essential for timely diagnosis and appropriate treatment, reducing misdiagnosis and antibiotic misuse. However, these tools are not consistently available in Italian PEDs. The objective of this survey is to assess the availability of diagnostic tools and current diagnostic practices in Italian PEDs, focusing on infection biomarkers, Point-of-Care (PoC) technologies, and the presence of disease-specific antimicrobial stewardship protocols.

**Methods:**

A cross-sectional survey, in collaboration with the Italian Society of Paediatric Emergency Medicine (SIMEUP), was conducted among clinicians working in PEDs across Italy. An online questionnaire was submitted at one referent for each centre, collecting data about each hospital activity, availability of diagnostic biomarkers, the use of PoC technologies, microbiological test reporting times, and availability of disease-specific protocols.

**Results:**

Data were collected from 55 PEDs representing almost all Italian regions, with a predominance of first- and second-level centres. Venous blood sampling was the primary method for investigating febrile children (80%), while only 20% of centres used capillary sampling as the initial diagnostic method. Limited access to PoC technologies for capillary samples (blood count and C-reactive protein available in 27.3%) contributed to this preference. Except for the SARS-CoV-2 nasopharyngeal swab (85% of PEDs), other PoC microbiological tests were less frequently available, making rapid etiological diagnosis more difficult. Laboratory-based microbiological tests were widely available (blood cultures, 93%; culture testing on specific samples, 84%; nasal swabs for influenza, 84%), but they often had reporting times exceeding 48 hours, which delays clinical decisions. Only 3.6% of centres had formal antibiotic prescription and de-escalation protocols, possibly leading to variability in clinical practice and overuse of antibiotics.

**Conclusions:**

This study highlights the fragmentation of diagnostic resources and practices in Italian PEDs, particularly in first-level centres. There is a clear need to expand access to PoC technologies, reduce test reporting times, and implement standardised protocols to improve diagnostic accuracy and enhance antibiotic stewardship.

## Background

Infectious diseases represent a leading cause of visits to Paediatric Emergency Department (PED), with fever as the most prevalent feature. They are characterised by significant clinical variability in both presentation and severity, making the diagnostic process challenging for clinicians, especially in the paediatric setting [1, 2]. Rapid and accurate diagnosis, based on shared and standardised protocols, is essential to avoid misdiagnosis that may result in complications or inadequate treatments. In this context, infection biomarkers and rapid etiological tests are fundamental tools to guide the clinician’s decision, especially when integrated into standardised clinical pathways and/or in antimicrobial stewardship disease-specific protocols [3–9].

To date, the perfect infection biomarker has not yet been identified. In recent years, an increasing number of host protein and microbiological point-of-care test have been developed and implemented in the PED [8, 10–15]. However, their implementation in standard practice varies significantly across Italian PED, reflecting a lack of standardisation and policy guidelines. This variability may lead to inaccurate assessments, inefficient resource use, and unnecessary antibiotic prescriptions [8, 16].

The aim of this study is to describe current diagnostic approaches for infectious diseases in Italian PEDs, with a focus on the availability and use of infection biomarkers, diagnostic practices, and antimicrobial stewardship protocols. Our goal is to identify major gaps and inform future interventions.

## Methods

A cross-sectional digital survey, with the collaboration of the Italian Society of Paediatric Emergency Medicine (SIMEUP), was conducted between September and December 2024 among clinicians working in Italian PEDs. The survey included questions designed to collect data on the types of diagnostic tests available, the use of Point-of-Care (PoC) technologies, the reporting time of microbiological tests, disease-specific protocols, and antibiotic stewardship practices. Additionally, we collected data on geographical distribution of participating centres, their level (first-, second-, od third level) and annual number of visits.

The survey was electronically distributed to a single referent for each centre. All responses were automatically recorded in Google Forms and exported to Microsoft Excel® for data management and initial processing. Data analysis was conducted using IBM SPSS Statistics 30.0.

According to Italian regulations, cross-sectional surveys do not require approval by an Ethics Committee, as no patient information is shared.

## Results

**Fig. 1:**
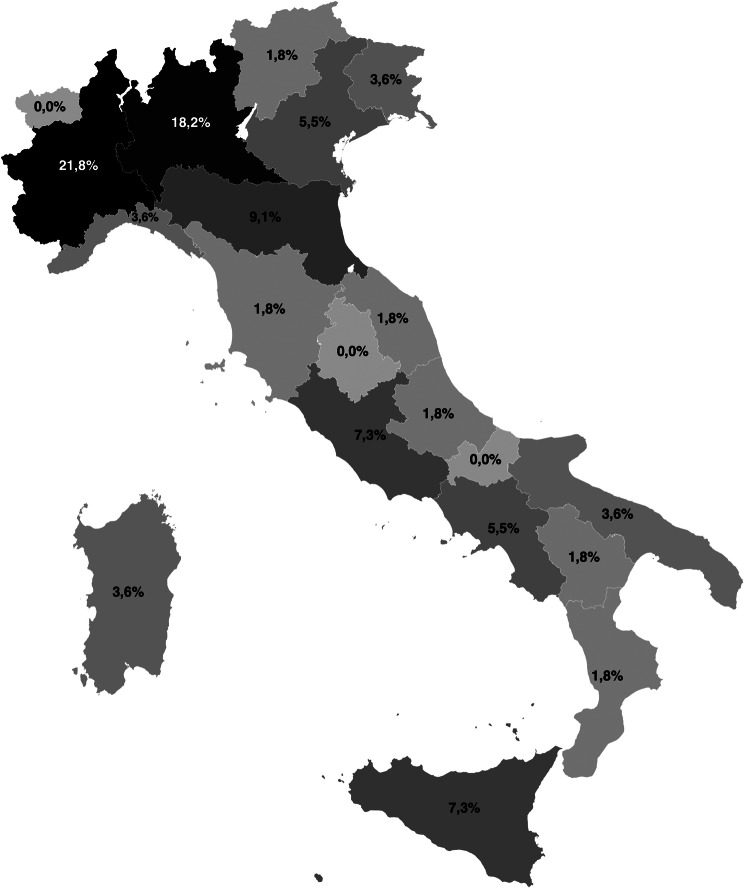
National distribution of survey responses (*n* = 55)

A total of 55 (response rate 68,75%) PEDs from nearly all Italian regions (17/20) completed the survey, with a predominance of Northern Italy (63.6%) compared to Central and Southern Italy (12.7% and 23.6%, respectively) (Fig. 1). Most facilities were first- and second-level hospitals, accounting for 65.5% of our sample. The remaining were third-level centres in paediatric hospitals; among them, the majority (82.3%) were university hospitals. Consequently, our sample predominantly consists of small PEDs accounting for < 20,000 annual visits, only 7.3% of the analysed centres reported > 40,000 visits/year.

Venous sampling remained the predominant method for investigating children with suspected infections, both at the time of diagnosis and during reassessment, while capillary sampling is available only in 20% of centres.

**Fig. 2:**
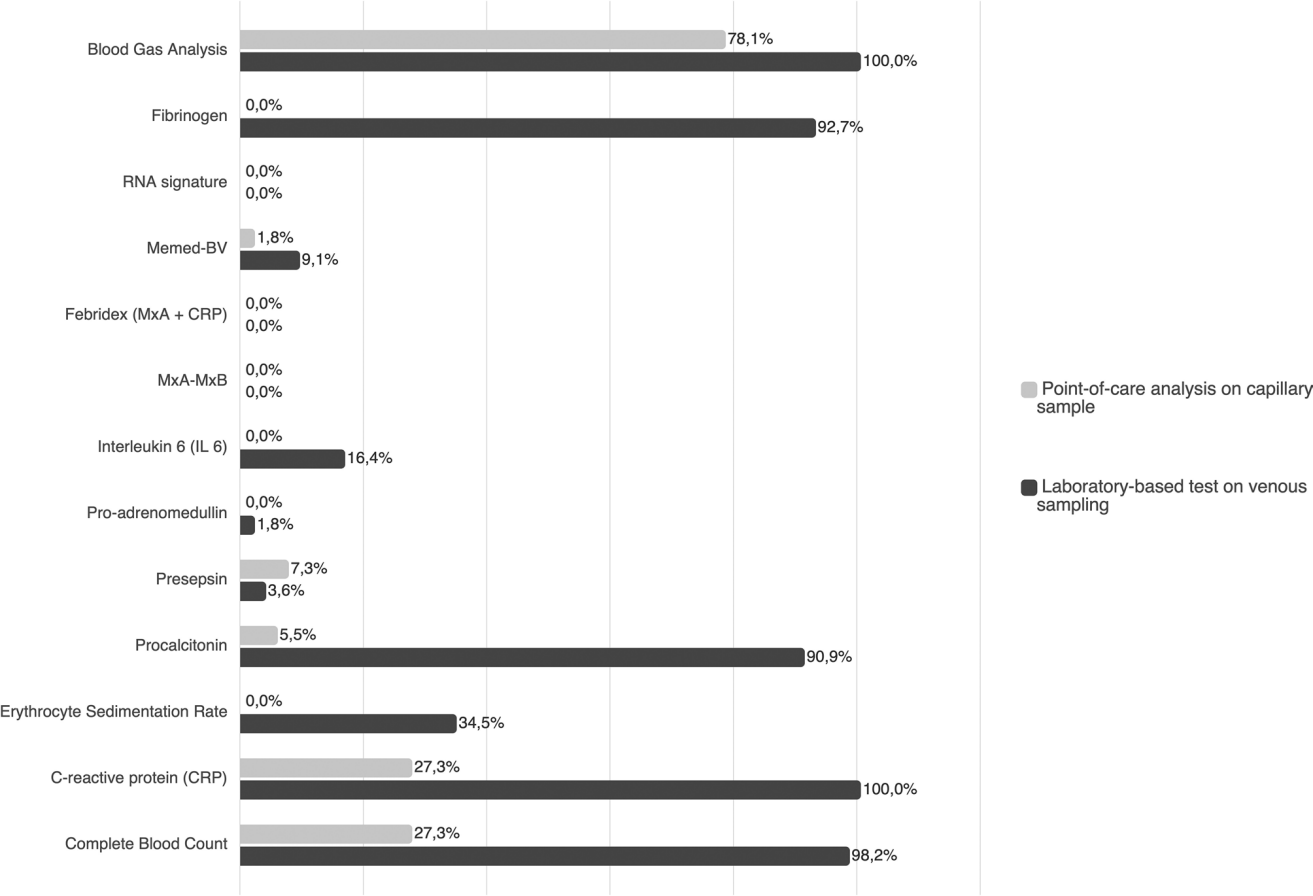
Availability of laboratory-based and point-of-care (PoC) biomarkers in Italian paediatric emergency departments (*n* = 55). Availability of laboratory-based and point-of-care (PoC) biomarkers in Italian paediatric emergency departments (PEDs). Grey bars represent point-of-care (PoC) tests performed on capillary samples, while black bars indicate laboratory-based tests performed on venous sample. Values are expressed as the percentage of PEDs (out of 55 centres) reporting access to each biomarker. The figure shows that some biomarker (e.g., complete blood count, CRP, fibrinogen) are widely available in laboratory settings, whereas point-of-care availability remains limited ( < 30% for most biomarker)

As shown in Table 1a, Table 1b and in Fig. 2, a disparity in the availability and in the use of diagnostic methods across was identified. About availability, despite blood gas analysis is available PoC in 78,1% of centres, only 27% of centres are equipped with PoC complete blood count and C-reactive Protein (CRP) analyser for capillary samples. As shown in Fig. 2, even fewer PEDs offered procalcitonin (5.5%), presepsin (7.3%) or Memed BV score (1,8%), a recently developed multiple host-biomarker that shown high diagnostic performance in distinguish between bacterial and viral infections.

**Table 1a:**
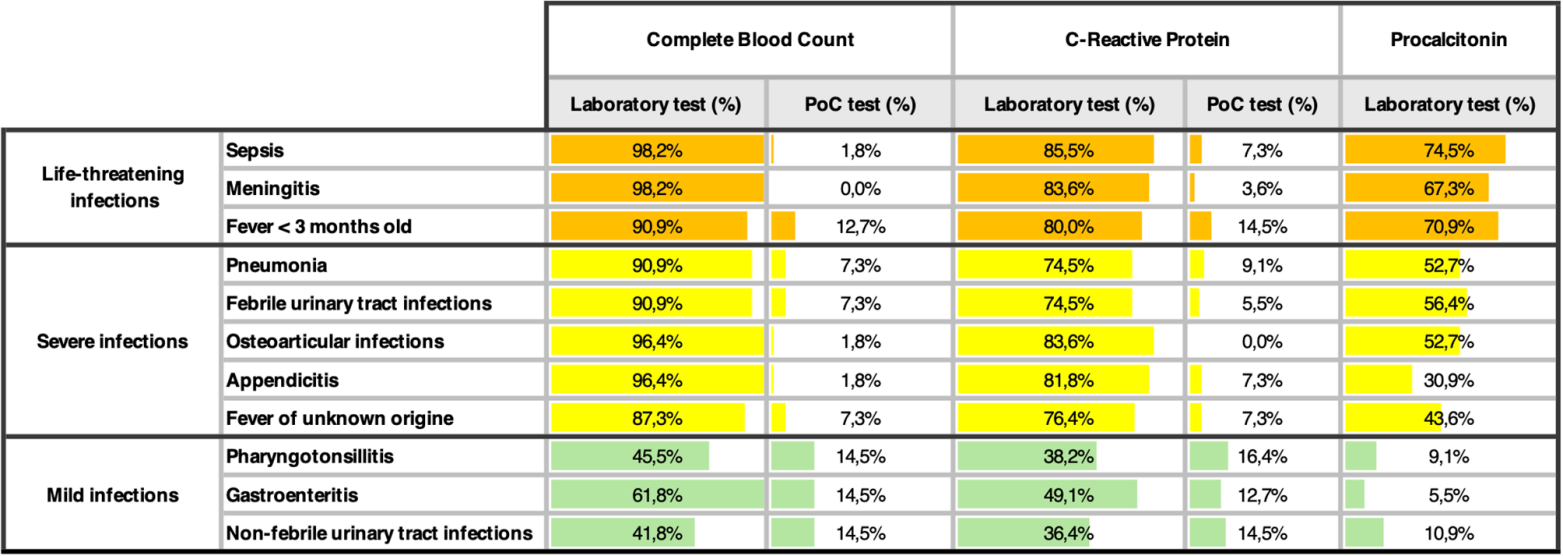
Use of complete blood count (CBC), C-Reactive protein (CRP), and procalcitonin as infection biomarkers at diagnosis in different infectious diseases by Italian PEDs. For CBC and CRP, the percentage of use of laboratory analysis on venous sampling versus point-of-care (PoC) analysis on capillary sampling is reported. Percentages calculated out of the total number of centres participating in the survey (*n* = 55).

**Table 1b:**
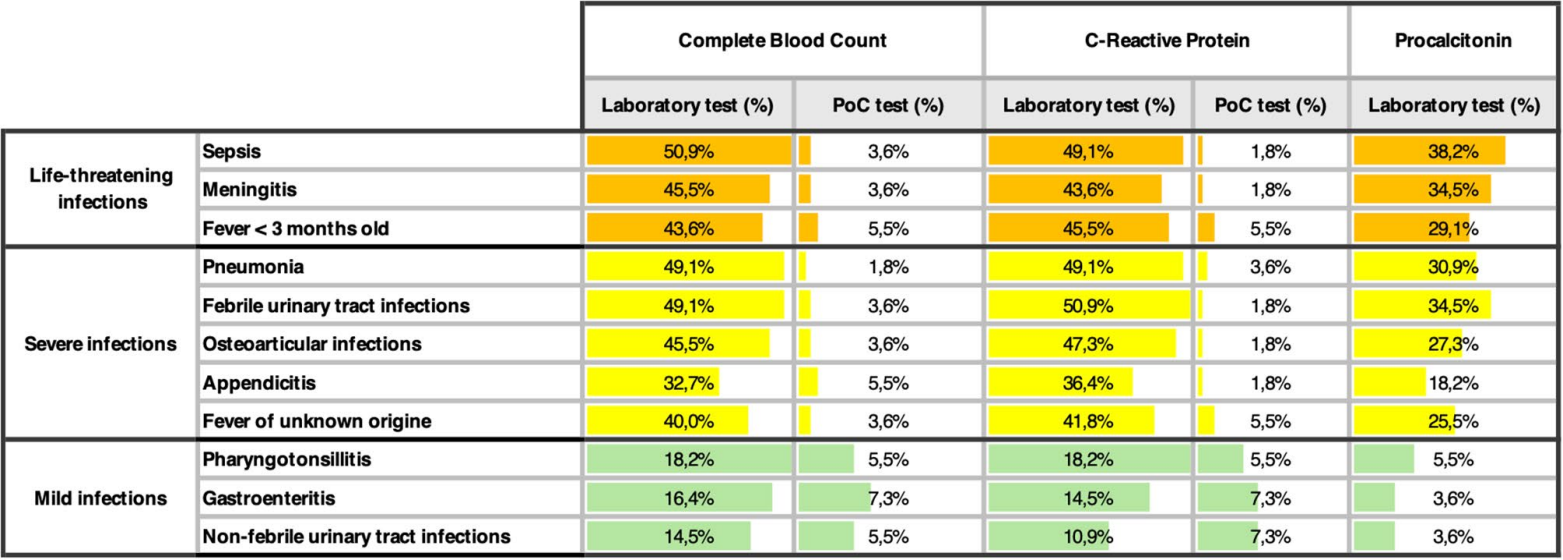
Use of complete blood count (CBC), C-Reactive protein (CRP), and procalcitonin as infection biomarkers during the diagnostic-therapeutic reassessment at 48 hours in different infectious diseases by Italian PEDs. For CBC and CRP, the percentage of use of laboratory analysis on venous sampling versus point-of-care (PoC) analysis on capillary sampling is reported. Percentages calculated out of the total number of centres participating in the survey (*n* = 55).

In contrast, nearly all PEDs could perform infection biomarkers on venous samples 24 hours a day, seven days a week (Fig. 2). In detail, complete blood count, CRP, procalcitonin, and fibrinogen were available in nearly the totality of centres, while interleukins and newly introduced biomarkers (Memed BV, presepsin and proadrenomedullin) were prerogative of third-level/university hospitals.

About biomarkers usage, the most employed were complete blood count, CRP and procalcitonin, obtained through venous sampling, for almost all cases, especially in potentially life-threatening or severe diseases. Regardless of the diagnosis, the 48–72 hours reassessment was always performed through venous sampling, as showed in Table 1b. Capillary sampling is used, when available, as first-approach methods in not severe diseases (e.g. tonsillitis, gastroenteritis, afebrile urinary tract infections) (Table 1a and Table 1b).

**Fig. 3:**
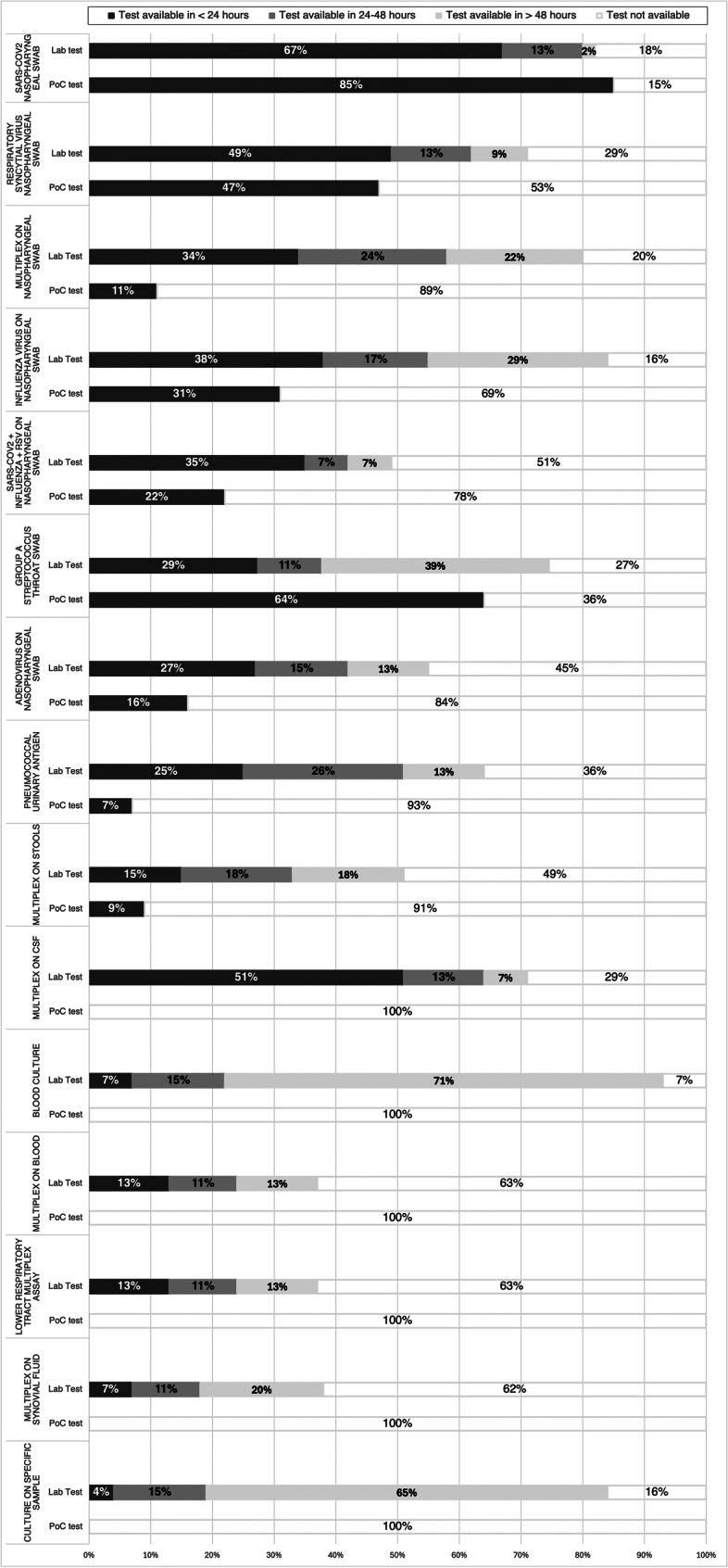
Availability and turnaround times of rapid etiological point-of-care (PoC) and laboratory microbiological tests in Italian paediatric emergency departments (PEDs) (*n* = 55 centres). Laboratory-based tests refer to standard analyses performed in central laboratories, while PoC tests are rapid assays performed directly in PEDs. The colour of the bars indicates the turnaround time: black bars represent results available in less than 24 hours, dark grey bars results within 24 to 48 hours, light grey bars results in more than 48 hours, and white bars indicate tests that are not available. Percentages refer to the proportion of PEDs reporting access to each test out of the total number of centres participating in the survey (*n* = 55). The figure shows that PoC tests allow faster reporting, while many laboratory-based methods, particularly cultures and multiplex assays, are often associated with longer delays exceeding 24 to 48 hours

About microbiological testing using PoC methodology, the most widely available tests were nasopharyngeal swabs for SARS-CoV-2 (85%), throat swabs for Group A Streptococcus (64%), and nasal swabs for Respiratory Syncytial Virus (RSV) (47%). Other respiratory pathogen tests, whether individual or multiplex, were less commonly available (Fig. 3). PoC investigations on stools and pneumococcal urinary antigen were available in less than 10% of centres.

Data about standard microbiological laboratory investigations and the related turnaround times for reporting are shown in Fig. 3. Blood culture was the most widely available test (93% of centres), but results typically took over 48 hours in 71% of cases. Similarly, 84% of centres offered culture testing on other clinical samples, with delayed reporting ( > 48 hours) in 65%.

Nasopharyngeal swabs for respiratory pathogens were available in varying proportions. The most common were tests for influenza (84%), SARS-CoV-2 (82%), and multiplex panels covering over 10 pathogens (80%) (Fig. 3). These swabs were also the microbiological tests most often reported within 24 hours.

Multiplex testing on cerebrospinal fluid (CSF) was available in 71% of centres, with 51% receiving results within 24 hours. In contrast, multiplex testing on stool, lower respiratory tract samples, joint fluid, blood, and pneumococcal urinary antigen was less frequently available. Reporting times for these are shown in Fig. 3.

Finally, only 3.6% had formal protocols for antibiotic de-escalation. In 5.5% of centres, therapeutic reassessment was performed through antimicrobial stewardship consultations without specific protocols. In the remaining 90.8%, no formal protocol were available: in detail, 56.3% followed informal practices, while 34.5% did not perform de-escalation at all.

## Discussion

This nationwide survey highlights a fragmented reality across Italian PEDs, predominantly composed of first- and second-level centres, with significant variability of available diagnostic resources. The majority of PEDs in this study were indeed small centres with less than 20,000 annual admissions and limited access to advanced diagnostic tools, particularly PoC technologies. Despite a moderate number of participants, our sample provides a representative overview of the Italian healthcare system. While the geographic distribution of hospitals reflects a widespread and accessible network, it also underscores the scarcity and variability of diagnostic resources across centres [17, 18].

Among PoC infection biomarkers, complete blood count and CRP are the most used. Such tests have been extensively studied and show excellent concordance with laboratory-based values [19, 20]. PoC tests using capillary sampling may offer several advantages in the ED setting. Their feasibility, rapid reporting time, and lower cost make them highly valuable in paediatric emergency care. They facilitate rapid diagnosis, reduce the children’s length of stay at the ED, and minimise the discomfort associated with more invasive procedures [19, 21, 22]. Despite these advantages, our survey revealed that only 20% of Italian centres use capillary sampling as the initial diagnostic test for febrile children. Limited availability of such tools largely explains the predominant reliance on venous sampling, even for conditions where less invasive methods would be reasonable. For instance, venous sampling remains the most common approach not only for severe infections such as sepsis, meningitis, and pneumonia, but also for milder conditions like pharyngotonsillitis, gastroenteritis, and afebrile Urinary Tract Infections (UTIs), where blood tests are usually less necessary, and capillary sampling could be a viable alternative (Table 1a and Table 1b). This reliance likely reflects both limited access to capillary PoC tools and a persistent reliance on venous testing. However, this approach might result in overburdening laboratory resources, increasing patient discomfort, and delaying clinical decisions. Expanding capillary PoC tools availability would address this gap and streamline diagnostic workflows, particularly in smaller PEDs.

Achieving an etiological diagnosis is another critical step in managing children with suspected infections. It simplifies the differential diagnostic process, reduces the need for additional diagnostic tests, and prevents inappropriate antibiotic prescriptions [4, 8, 13, 14, 23]. This is especially relevant for respiratory tract infections, which are among the leading causes of PEDs visits and the most common reason for antibiotic prescriptions, despite their mainly viral aetiology [4, 5, 8, 13, 24–26]. The availability of rapid etiological or laboratory-based tests with short reporting times is essential in this setting. Nevertheless, their limited access represents a significant critical finding of our survey.

Laboratory-based tests such as blood cultures and other microbiological cultures are widely available in Italian PEDs, but their results often need more than 48 hours (Fig. 3), likely delaying crucial therapeutic decisions. Only nasopharyngeal swabs for SARS-CoV-2 are both widely accessible (82%) and processed rapidly (within 24 hours in 67% of centres), reflecting the prioritisation of COVID-19 diagnostics during the pandemic. In contrast, the availability of tests for other common pathogens, such as Adenovirus or extended respiratory and gastrointestinal panels, is still extremely limited (Fig. 3).

Multiplex real-time polymerase chain reaction (PCR) on cerebrospinal fluid (CSF) represents another critical issue: only 71% of Italian centres have access to this diagnostic tool. A rapid clinical and etiological diagnosis of central nervous system infections is the cornerstone for appropriate targeted therapy that will allow better clinical outcomes. In this context, multiplex PCR panels on CSF could provide reliable results in a significantly shorter time compared to culture-based methods [23, 27]. Because of the severity of these infections with time-dependent outcomes, multiplex PCR panels on CSF are desirable in nearly all healthcare facilities.

PoC etiological tests represent another valuable diagnostic tool, providing rapid results with sensitivity and specificity comparable to laboratory-based real-time PCR, especially for respiratory viruses [13–15]. Nevertheless, our results suggest that only nasopharyngeal swabs for SARS-CoV-2 are widely available, whereas other rapid etiological tests are available in a minority of centres. This disparity highlights how the recent pandemic reshaped diagnostic priorities: while COVID-19 was granted universal coverage, other pathogens continue to be underdiagnosed owing to limited testing, which contributes to delays.

Adenovirus infections exemplify the diagnostic challenges resulting from limited access to comprehensive etiological testing. Their clinical and laboratory features can closely mimic serious conditions such as bacterial infections or Kawasaki disease [28], often leading to unnecessary hospitalisations or inappropriate treatments. Integrating Adenovirus testing into routine diagnostics could improve diagnostic accuracy, optimise clinical management, and reduce the use of unnecessary healthcare resources.

Pharyngitis is another example that underscores the importance of etiological testing, particularly when incorporated into standardised clinical protocols. Because Group A β-haemolytic Streptococcus cannot be clinically distinguished from viral pharyngitis, this condition is one of the leading causes of antibiotic misuse in children, especially in settings where diagnostic swabs are unavailable [26, 29, 30]. Evidence consistently shows that rapid antigen detection tests reduce inappropriate antibiotic prescriptions, with sensitivity rates of about 85% when used within standardised clinical pathways [9, 13]. Nevertheless, only 64% of the PEDs in this survey reported access to these tests. This aligns with findings by Milani et al., who observed that around 50% of Italian PEDs use rapid tests for streptococcal pharyngitis, often without combining them with clinical scoring systems [29].

The absence of validated protocols further aggravates diagnostic challenges in Italian PEDs. Several studies highlight that adopting clinical practice protocols can improve diagnostic accuracy and reduce unnecessary antibiotic prescriptions, particularly for pharyngitis and respiratory tract infections [5–9, 29]. However, only a minority of Italian PEDs have established protocols for antibiotic de-escalation or standardised diagnostic pathways. Most centres instead rely on either informal practices or ad hoc consultations. This lack of standardisation increases variability in clinical practice, wastes resources, delays care, and promotes the overuse of broad-spectrum antibiotics, thereby contributing to antimicrobial resistance. Developing, implementing and sharing validated protocols across all PEDs, along with training programs and antimicrobial stewardship initiatives, is essential to ensure consistent, evidence-based care [5, 8].

Our study is not without limitations. The voluntary participation in the survey could introduce a selection bias, with higher participation from larger centres and those more engaged with the scientific community, compared to smaller centres. As a result, our findings may overrepresent hospitals with greater diagnostic resources and higher awareness of the topic, while potentially underestimating the challenges faced by smaller or less equipped centres. This aspect could limit the overall generalizability of our analysis and should be taken into account when interpreting the results.

On the other hand, the participation of a wide range of centres across the country ensures a nationally representative overview, and the observed heterogeneity itself underscores the importance of this issue. We believe that highlighting such variability provides a valuable starting point for further multicentre studies specifically designed to address the representativeness of different settings. To our knowledge, this is the first study to analyse these aspects within the Italian setting.

## Conclusions

Our study highlights the fragmented nature of paediatric emergency care on infectious diseases in Italy, characterised by significant variability of available diagnostic resources and the lack of standardised protocols. To address these gaps, we suggest some possible strategies to implement diagnostic technologies and protocols in a first line setting such as the PED.

First, national health authorities should promote the progressive implementation of capillary PoC technologies, with particular attention to smaller centres and resource-limited settings. This could be facilitated through national coordination plans, incentives for innovation, and the integration of PoC devices into existing clinical pathways.

Second, to reduce reporting times for microbiological tests, efforts should focus on both organisational and technological interventions: centralised laboratory networks with streamlined workflows, the adoption of rapid molecular diagnostic platforms, and the integration of electronic reporting systems to ensure timely communication of results to clinicians.

Third, there is an urgent need to develop and disseminate national, evidence-based diagnostic and therapeutic protocols, tailored to paediatric emergency care. This process should involve multidisciplinary expert panels and professional societies, and be followed by multicentre quality improvement initiatives to ensure adoption and adherence across different settings.

**Fig. 4:**
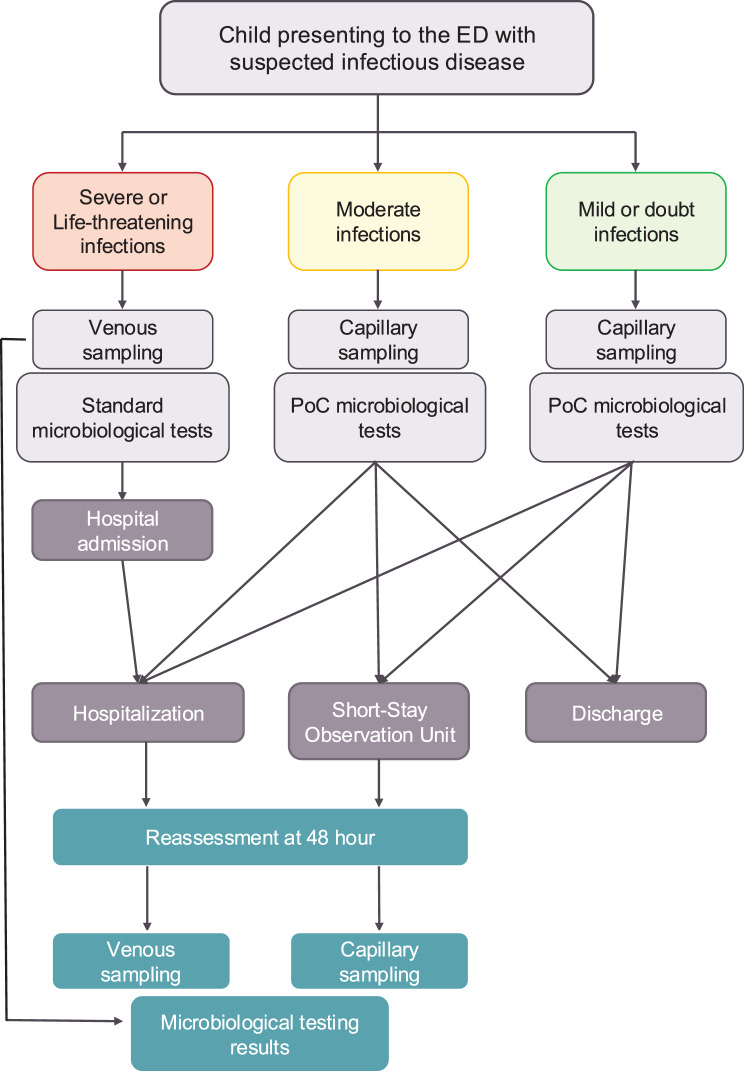
Simplified diagnostic pathways for managing infectious diseases in Italian paediatric emergency departments. Flow chart illustrating the main diagnostic approaches adopted in Italian paediatric emergency departments (PEDs), according to disease severity and availability of diagnostic resources. Severe or life-threatening infections are generally managed with venous sampling and hospital admission, whereas moderate infections may involve venous or capillary sampling with microbiological tests. For mild or uncertain infections, point-of-care (PoC) tests on capillary samples are preferred when available, facilitating early discharge or observation. Reassessment at 48 hours is usually performed with venous or capillary sampling, integrating microbiological test results when available. This model provides a simplified representation of current practices and highlights areas for future standardisation and educational use

These three complementary measures can reduce inter-centre variability, improve patient outcomes and reduce antibiotic prescriptions. Figure 4 summarises the proposed diagnostic pathways, offering a structured framework that may facilitate clinical decision-making, enhance standardisation, and serve as an educational tool within paediatric emergency care. The COVID-19 pandemic demonstrated that when healthcare systems recognise a priority, they can adapt and restructure accordingly. It is time to apply this same urgency to the growing issue of antimicrobial resistance in children.

## Data Availability

The datasets used during the current study are available from the corresponding author on reasonable request.

## Abbreviations

CBC: Complete Blood Count
CRP: C-reactive Protein
CSF: Cerebrospinal Fluid
PED: Paediatric Emergency Department
PoC: Point-of-Care
PCR: Polymerase Chain Reaction
RSV: Respiratory Syncytial Virus
SIMEUP: Italian Society of Paediatric Emergency Medicine
UTIs: Urinary Tract Infections
